# Experience from Asian centers in a named-patient-use program for afatinib in patients with advanced non-small-cell lung cancer who had progressed following prior therapies, including patients with uncommon *EGFR* mutations

**DOI:** 10.1007/s10147-021-01869-0

**Published:** 2021-03-30

**Authors:** Gee-Chen Chang, David Chi-Leung Lam, Chun-Ming Tsai, Yuh-Min Chen, Jin-Yuan Shih, Shyam Aggarwal, Shuhang Wang, Sang-We Kim, Young-Chul Kim, Ibrahim Wahid, Rubi Li, Darren Wan-Teck Lim, Virote Sriuranpong, Raymond Tsz-Tong Chan, Robert M. Lorence, Philippe Carriere, Christina Raabe, Agnieszka Cseh, Keunchil Park

**Affiliations:** 1 Division of Chest Medicine, Department of Internal Medicine, Taichung Veterans General Hospital, No. 1650, Section 4, Taiwan Boulevard, Xitun District, Taichung, Taiwan; 2 Division of Pulmonary Medicine, Department of Internal Medicine, Chung Shan Medical University Hospital, Taichung, Taiwan; 3 Institute of Medicine and School of Medicine, Chung Shan Medical University, Taichung, Taiwan; 4 Queen Mary Hospital, The University of Hong Kong, Hong Kong, China; 5 Taipei Veterans General Hospital, Taipei, Taiwan; 6 Department of Chest Medicine, Taipei Veterans General Hospital, and School of Medicine, National Yang-Ming Medical University, Taipei, Taiwan; 7 National Taiwan University Hospital, Taipei, Taiwan; 8 Sir Ganga Ram Hospital Rajinder Nagar, New Delhi, India; 9 Beijing Cancer Hospital, Beijing, China; 10 Asan Medical Center, University of Ulsan College of Medicine, Seoul, Republic of Korea; 11 Chonnam National University, Hwasun Hospital, Jeonnam, Republic of Korea; 12 Beacon International Specialist Centre, Selangor, Malaysia; 13 St Luke’s Medical Center, Quezon City, Philippines; 14 National Cancer Centre, Singapore, Singapore; 15 Chulalongkorn University and the King Chulalongkorn Memorial Hospital, Pathumwan, Bangkok, Thailand; 16 Hong Kong Pacific Centre, Kowloon, Hong Kong, China; 17 Boehringer Ingelheim Pharmaceuticals, Inc., Ridgefield, CT USA; 18 Boehringer Ingelheim International GmbH, Ingelheim Am Rhein, Germany; 19 Boehringer Ingelheim RCV GmBH & Co. KG, Vienna, Austria; 20 Samsung Medical Center, Sungkyunkwan University School of Medicine, Seoul, Republic of Korea

**Keywords:** Afatinib, *HER2* mutations, Lung cancer, Named patient use, NSCLC, Uncommon *EGFR* mutations

## Abstract

**Background:**

This study evaluated outcomes among patients with advanced/metastatic non-small-cell lung cancer (NSCLC) treated at Asian centers participating in the global named-patient-use (NPU) program for afatinib.

**Methods:**

Patients had progressed after initial benefit with erlotinib or gefitinib, and/or had an *EGFR* or *HER2* mutation, had no other treatment options, and were ineligible for afatinib trials. The recommended starting dose of afatinib was 50 mg/day. Dose modifications were allowed, and afatinib was continued as long as deemed beneficial. Response and survival information was provided voluntarily. Safety reporting was mandatory.

**Results:**

2242 patients (26% aged ≥ 70 years, 96% with adenocarcinoma) received afatinib at centers in 10 Asian countries. Most were heavily pre-treated, including prior treatment with erlotinib or gefitinib. Of 1281 patients tested, 1240 had *EGFR* mutations (common: 1034/1101; uncommon: 117/1101). There were no new safety signals, the most common adverse events being rash and diarrhea. Objective response rate (ORR) was 24% overall (*n* = 431 with data available), 27% for patients with common *EGFR* mutations (*n* = 230) and 28% for those with uncommon mutations (*n* = 32); median time to treatment failure (TTF) in these groups was 7.6 months (*n* = 1550), 6.4 months (*n* = 692) and 8.4 months (*n* = 83), respectively. In patients with *EGFR* exon 20 insertions (*n* = 23) and *HER2* mutations (*n* = 12), median TTF exceeded 12 months.

**Conclusions:**

Patient outcomes in this study were similar to those reported in the analysis of the global NPU. Afatinib achieved clinical benefits in patients with refractory NSCLC. ORR and TTF were similar between patients with tumors harboring uncommon and common *EGFR* mutations.

## Introduction

Afatinib is an irreversible inhibitor of the ErbB receptor family (EGFR [epidermal growth factor receptor]/ErbB1; HER2 [human epidermal growth factor receptor 2]/ErbB2; and HER4/ErbB4). In contrast to the first-generation EGFR tyrosine kinase inhibitors (TKIs) erlotinib and gefitinib, which bind reversibly to the ErbB1 receptor, afatinib covalently binds to all ErbB family receptors, blocking signaling and causing sustained inhibition of mitogenic activity [1, 2]. Afatinib is approved in the European Union, USA, Canada, Switzerland, Australia, and several Asian, Latin American, and Middle Eastern countries as an oral, once-daily tablet for patients with non-small-cell lung cancer (NSCLC) and activating *EGFR* mutations. In addition to the common mutations, exon 19 deletions (del19) and L858R substitutions, there is evidence that afatinib is active against some uncommon *EGFR* mutations, including L861Q, G719X, and S768I [3].

A global named-patient-use (NPU) program for afatinib was initiated in Germany and Australia in May 2010, for patients with advanced or metastatic NSCLC who had progressed after clinical benefit during previous treatment with erlotinib or gefitinib and/or had an activating *EGFR*/*HER2* mutation, had exhausted all other treatments and were ineligible for an afatinib trial. The main objective of the program was to provide compassionate access to treatment for patients with no other established therapeutic options. The program continued until January 2016, by which time a total of 5636 patients had been treated in 49 countries on six continents. In an analysis of treatment outcomes in 3966 patients from 41 countries (excluding Taiwan), the median time to treatment failure (TTF) was 4.4 months and the objective response rate (ORR) was 23% [4]. Outcomes of patients treated in centers in specific countries [5–8], and with *HER2* mutations [9] have also been described.

Here we present an analysis of treatment outcomes in patients who were treated at centers in 10 Asian countries. The large size of the NPU program made it possible to evaluate treatment outcomes in patients with both common and uncommon *EGFR* mutations. Understanding the influence of *EGFR* mutations is particularly important for patients in Asian countries, as *EGFR* mutations are prevalent among patients from this region [10].

## Materials and methods

The design of the NPU has been reported previously [4]. Key details are summarized below.

### Patients

Patients were eligible if they had advanced/metastatic NSCLC, had progressed after initially achieving clinical benefit (complete response [CR], partial response [PR], or stable disease [SD] lasting at least 6 months) during treatment with erlotinib or gefitinib and/or had an activating *EGFR* mutation or a *HER2* mutation, had exhausted other treatment options and were ineligible to participate in an afatinib trial. Previous TKI therapy was not mandatory for all patients with confirmed mutations. Chemotherapy-naïve patients were eligible for inclusion if they were unfit to receive chemotherapy and were deemed ineligible to participate in an actively recruiting afatinib trial. The NPU program procedures (including enrollment criteria and treatment details) were adapted locally and approved in each region according to local regulations. The current analysis was conducted using data collected for patients treated at centers in Asian countries only.

### Afatinib dose

The recommended starting dose of afatinib was 50 mg/day, as used in the phase III LUX-Lung 1 study of afatinib following failure of prior erlotinib/gefitinib [11]. Lower starting doses (40 or 30 mg/day) were allowed at the discretion of the treating physician. Tolerability-guided dose modifications were also allowed, using 10-mg steps to a maximum of 50 mg/day and a minimum of 30 mg/day. Afatinib was continued as long as deemed beneficial by the treating physician.

Enrollment into the NPU program was terminated within each country once afatinib became commercially available locally; enrollment had ceased worldwide by January 2016. In some countries, patients were switched to commercially available afatinib provided by Boehringer Ingelheim; in others, patients continued to receive afatinib via the NPU program. In both cases, afatinib was continued as long as treatment was deemed beneficial by the treating physician.

### Outcome measures

TTF was defined as the time from the date of initiation of afatinib to the date of discontinuation, switch to another drug, death, or the last available data, whichever occurred first. The ORR was defined as the proportion of patients with a recorded response outcome (CR, PR, SD, progressive disease [PD], or mixed response) who achieved a CR or PR. The disease control rate (DCR) was defined as a proportion of patients with response information available who achieved a CR, PR, or SD. There was no independent radiologic verification of responses, SD or PD.

Physicians were required to report safety-related information including all adverse events (AEs) leading to discontinuation of afatinib or deemed to be related to afatinib by the treating physician, and all serious AEs.

### Data capture and analysis

Data collected during the program were provided voluntarily by the participating physicians and only safety reporting was mandatory.

No site monitoring, site audits, data cleaning, or structured data collection was conducted, except for the use of standard serious AE forms and reporting. Data were analyzed using SAS® software (Version 9.4, SAS Institute, Inc., NC, USA). The cut-off date for data analysis was January 18, 2016.

Baseline demographics and outcomes during treatment with afatinib were analyzed. Subgroup analyses were also conducted, including analyses of groups defined by the presence or absence of common and uncommon *EGFR* mutations, specific *EGFR* mutations, and *HER2* mutations.

### Ethics

All procedures were approved by the responsible ethics committees and the required country-specific and regional regulatory authorities were informed about the program.

The datasets generated and analyzed during the NPU program are available from the corresponding author on reasonable request.

## Results

*Patient* *characteristics and treatment*

As of the cut-off date, 2242 patients with NSCLC had received afatinib at treatment centers across 10 Asian countries (Table 1); 26% were aged ≥ 70 years. Of the 1924 patients with known tumor histology, 96% had adenocarcinoma. Most patients were heavily pre-treated; of the 2242 patients treated with afatinib, 2223 had received at least one line of previous therapy and 2202 had been treated with erlotinib and/or gefitinib. Sixty-two percent had previously received at least two lines of chemotherapy and 65% had received at least three lines of systemic therapy (Table 2). Nineteen patients who were ineligible for chemotherapy received first-line afatinib at the request of the treating physicians on the basis that they considered it of potential benefit.

**Table 1:** Baseline demographic and clinical characteristics

Characteristic, *n* (%)	*N* = 2242
Country	
Eastern Asia	
Taiwan^a^	840 (37.5)
Republic of Korea	377 (16.8)
Hong Kong	302 (13.5)
Philippines	154 (6.9)
Singapore	145 (6.5)
Thailand	108 (4.8)
Malaysia	96 (4.3)
China	48 (2.1)
Indonesia	18 (0.8)
Non-Eastern Asia	
India	154 (6.9)
Gender^b^	*N* = 2220
Male	896 (40.4)
Female	1324 (59.6)
Age^b^, years	*N* = 2192
Median	61
25^th^ percentile	53
75^th^ percentile	70
70–80, *n* (%)	444 (20.3)
≥ 80, *n* (%)	120 (5.5)
Histology^b^	*N* = 1924
Adenocarcinoma	1853 (96.3)
Squamous cell carcinoma	21 (1.1)
Large cell carcinoma	3 (0.2)
Other	47 (2.4)
Starting dose of afatinib^b^, mg/day	*N* = 1333
50	609 (45.7)
40	631 (47.3)
30	93 (7.0)

Data cut: January 18, 2016

*NPU* named-patient-use

^a^Patients from Taiwan were omitted from a previous report on the global NPU program (Cappuzzo et al. 2018) [4] due to a misunderstanding

^b^Patients whose physicians provided relevant information

**Table 2:** Previous therapies

Therapy^a^	*N* = 2242
No previous treatment reported, *n* (%)	19 (0.8)
Any previous treatment, *n* (%)	2223 (99.2)
Previous lines of chemotherapy, median (IQR)	2 (1–3)
Previous lines of chemotherapy (%)	
≥ 3	32
≥ 2	62
1	23
0	15
Previous lines of systemic therapy, median (IQR)	3 (2–4)
Previous lines of systemic therapy (%)	
≥ 4	37
≥ 3	65
2	21
1	14
0	0
Previous TKI use	
Any TKI^b^, *n* (%)	2202/2223 (99.1)
Erlotinib and/or gefitinib	2202 (99.1)
Erlotinib only	866/2202 (39.3)
Gefitinib only	927/2202 (42.1)
Erlotinib and gefitinib	409/2202 (18.6)

*IQR* interquartile range, *TKI* tyrosine kinase inhibitor

^a^Patients whose physicians provided information on previous therapies

^b^Erlotinib, gefitinib, lapatinib, trastuzumab, or afatinib

Mutation status was reported for 1281/2242 patients (57%), 97% of whom were *EGFR* mutation-positive (Table 3). Among patients with a specified *EGFR* mutation, 94% had a common *EGFR* mutation and 11% had an uncommon *EGFR* mutation, including 47 patients with T790M, 45 patients with G719X, L861Q or S768I, and 35 patients with *EGFR* ex20ins (Table 3).

**Table 3:** Tumor *EGFR* and *HER2* mutation status

Mutation status, *n* (%)	
*EGFR*	*N* = 1281^a^
Mutation-positive	1240/1281 (96.8^b^)
Site of mutation specified	1101/1240 (88.8^c^)
Common *EGFR* mutations (del19 or L858R)	1034/1101 (93.9^d^)
Uncommon *EGFR* mutations (any)	117/1101 (10.6^d^)
T790M	47/117 (40.2^e^)
G719X, L861Q, S768I	45/117 (38.5^e^)
ex20ins	35/117 (29.9^e^)
Wild-type	41/1281 (3.2^b^)
Not reported	961
*HER2*	
Total^a^ *HER2* mutation-positive	12 (0.5^b^)
Also *EGFR* mutation-positive	0/12 (0^b^)
*HER2* mutation specified	7/12 (58^f^)
p.A775_G776insYVMA HER2	7/7 (100^ g^)

*del19* exon 19 deletions, *EGFR* epidermal growth factor receptor, *HER2* human epidermal growth factor receptor 2, *L858R* Leu858Arg point mutations in exon 21

^a^Patients whose physician volunteered information on the presence or absence of the specified mutations

The denominators used to calculate the percentage data were:

^b^Total number of patients with mutation status available

^c^Number of *EGFR* mutation-positive patients

^d^Number of patients with specified *EGFR* mutations

^e^Number of patients with specified uncommon *EGFR* mutations

^f^Number of *HER2* mutation-positive patients

^g^Number of patients with specified *HER2* mutations

Twelve patients were *HER2* mutation-positive, with no concurrent *EGFR* mutations. Seven had a specified *HER2* mutation, all being p.A775_G776insYVMA insertions at nucleotide 2325 (Table 3).

### Response to therapy, overall and according to mutation status

Information on response to afatinib was provided for 431 patients (19%), 78% of whom (335/431) had PR or SD; the ORR was 24% and the DCR was 78% (Table 4). Among patients with available information on both response and mutation status, ORR was 28% in patients with *EGFR* mutations (*n* = 267), 27% in those with common *EGFR* mutations (*n* = 230), and 28% in those with uncommon *EGFR* mutations (*n* = 32). In patients with G719X, L861Q, or S768I (*n* = 7), the ORR was 43% (Table 4). For patients with a specified *HER2* mutation, 4/7 had response information available; all four had disease control (PR or SD) and one of the four had a PR (Table 4). In patients with a < 6-month interval between discontinuing previous EGFR TKI treatment and initiating afatinib (n = 586), the ORR was 23.5% and the DCR was 78.2%. In those patients with a < 12 month interval (n = 986), the ORR and DCR were 25.5 and 78.2%, respectively.

**Table 4:** Response to afatinib

Reported response, *n* (%)	
Objective response rate (ORR)	105/431 (24.4)
Partial response	105 (24.4)^a^
Complete response	0
Mixed response	0
Stable disease	230 (53.4)^a^
Progressive disease	96 (22.3)^a^
Disease control rate	335 (77.7)^a^
ORR by mutation status	
*EGFR* mutation-positive^b^	74/267 (27.7)
Common *EGFR* mutations (del19 or L858R)	63/230 (27.4)
Uncommon *EGFR* mutations (any)	9/32 (28.1)
T790M	5/20 (25.0)
G719X, L861Q, S768I	3/7 (42.9)
ex20ins	1/5 (20.0)
*HER2* mutation-positive	1/7 (14.2)
p.A775_G776insYVMA	1/4 (25.0)
ORR by prior TKI use	
In patients with ≥ 2 years’ previous use of erlotinib or gefitinib	8/84 (9.5)
In patients with PR/CR during previous use of erlotinib	30/124 (24.2)

*CR* complete response, *del19* exon 19 deletions, *EGFR* epidermal growth factor receptor, *ex20ins* exon 20 insertions, *HER2* human epidermal growth factor receptor 2, *L858R* Leu858Arg point mutations in exon 21, *MR* mixed response, *ORR* objective response rate, *PR* partial response, *TKI* tyrosine kinase inhibitor

^a^Percentage response is based on the total number of patients with information available on the response to afatinib, as well as documented evidence of having received afatinib through the reported start date (*n* = 431)

^b^Mutation type was not known for two patients

### TTF, overall and according to mutation status and previous use of TKIs

Among the 1550 patients (69%) for whom TTF data were available, median TTF was 7.6 months (Table 5). Among those for whom information on both TTF and mutation status was available, median TTF was 7.2 months for patients with *EGFR* mutations (*n* = 834), 6.4 months for those with common *EGFR* mutations (*n* = 692) and 8.4 months for those with uncommon *EGFR* mutations (*n* = 83). In patients with G719X, L861Q, or S768I mutations (*n* = 28), median TTF was 7.8 months, but in those with *EGFR* ex20ins (*n* = 23) and *HER2* mutations (*n* = 12), median TTF exceeded 12 months.

**Table 5:** Time to treatment failure

	*n*	Median TTF months (IQR)
Overall^a^, *N*	1550	7.6 (2.6–24.3)
Mutation status		
*EGFR* mutation-positive	834	7.2 (2.5–22.6)
Specified *EGFR* mutation (common or uncommon)	740	6.5 (2.3–22.4)
Common *EGFR* mutations (del19 or L858R)	692	6.4 (2.3–22.4)
Uncommon *EGFR* mutations (any)	83	8.4 (1.9–22.4)
T790M	35	5.9 (1.9–10.8)
G719X, L861Q, S768I	28	7.8 (0.8–25.4)
ex20ins	23	18.9 (8.5–27.4)
*HER2* mutation-positive	12	12.2 (2.6–25.2)
p.A775_G776insYVMA	7	12.4 (4.0–15.8)
Prior TKI use		
Any previous use of erlotinib or gefitinib	922	8.7 (2.8–25.2)
≥ 2 years previous use of erlotinib or gefitinib	338	10.2 (3.5–26.5)
Any reported response (PR, CR, MR, SD, PD) during previous use of erlotinib	865	8.7 (2.8–24.7)
PR/CR reported during previous use of erlotinib	383	8.2 (2.6–23.5)

*CR*, complete response; *del19*, exon 19 deletion; *EGFR*, epidermal growth factor receptor; *ex20ins*, exon 20 insertions; *IQR*, interquartile range; *L858R* Leu858Arg point mutations in exon 21, *MR*, mixed response; *PD*, progressive disease; *PR*, partial response; *SD*, stable disease, *TKI*, tyrosine kinase inhibitor; *TTF*, time to treatment failure

^a^Patients whose physicians provided information on TTF, and documented evidence of having received afatinib through the reported start date

Median TTF for patients who had previously received erlotinib or gefitinib was 8.7 months, and 8.2 months for those who had previously responded (CR/PR) to erlotinib (Table 5). In patients with < 6-/ < 12-month intervals between the discontinuation of prior EGFR TKI treatment and initiating afatinib, median TTF was 8.4 and 7.6 months, respectively.

### Adverse events

The most frequently reported AEs were rash and diarrhea (Table 6).

**Table 6:** Most frequently reported AEs^a^ of any grade (≥ 1% of patients receiving afatinib)

AE	N
Rash/pruritus/dry skin/dermatitis acneiform/acne	536
Diarrhea	515
Stomatitis/mucosal inflammation	326
Paronychia	203
Decreased appetite	147
Nausea/vomiting	123
Fatigue/asthenia	78
Pneumonia	69
Pleural effusion	37
Cough	36
Pyrexia	35

The data shown are AE counts, not the numbers of patients with each AE. An individual patient could have several episodes or counts of a particular AE

The data are not shown as percentages because of the risk that this could be misunderstood as representing the percentage of patients with each AE, as a proportion of the total number of patients

*AE* adverse event

^a^Excludes malignant neoplasm progression and death

## Discussion

This analysis of patients from Asian countries involved in the afatinib NPU program revealed clinically meaningful ORRs and TTF in this heavily pretreated and resistant/refractory advanced NSCLC patient population. Ninety-seven percent of patients with information on *EGFR* mutation status were *EGFR* mutation-positive, whereas 93% of the global NPU population were *EGFR* mutation-positive (2407/2595 patients, excluding patients from Taiwan) [4].

The findings of this study were generally similar to those of an analysis of the global NPU population [4]. This is an important finding as, without evidence, it cannot be assumed that patients from Asia respond similarly to those from other regions. However, median TTF for *EGFR* mutation-positive patients was 7.2 months for the Asian centers, compared with 4.3 months for the global NPU program, while the ORRs were 28% and 25%, respectively. The disparity in median TTF may have been influenced by differences between regions in the rates of reporting of key end dates, such as end of treatment and date of death. The median TTF for patients in this analysis of data from Asian centers may also have been influenced by the particularly prolonged TTF of patients in Taiwan 14.2 months (n = 273) [12]. When the NPU program began, investigators/clinicians in Taiwan had already gained experience in the use of afatinib, through their involvement in phase I and II trials. As a result, they may have been able to optimize their use of afatinib, potentially through more successful management of side effects, thereby allowing patients to remain on treatment for longer and to achieve greater clinical benefit. In contrast, in many other countries, physicians’ first experience with afatinib was within the NPU.

The clinical benefits of afatinib appeared to be similar between patients with NSCLC tumors harboring common or uncommon *EGFR* mutations. The median TTF in the subgroup with uncommon *EGFR* mutations was 8.4 months, versus 6.4 months in the subgroup with common *EGFR* mutations; ORR was 28% and 27%, respectively. In the global NPU program, TTF was 4.3 months in both subgroups [4], while ORR was 26% and 25%, respectively [4].

In the current analysis, among the 28 patients with G719X, L861Q, or S768I mutations, median TTF was 7.8 months, compared with 4.7 months for patients with these mutations in the global NPU population (*n* = 77; [4]). The ORR in the current analysis was 43% (3/7 patients), compared with 30% (8/27 patients) in the global NPU analysis [4]. The difference in median TTF between patients with G719X, L861Q, or S768I mutations in the Asian subgroup and the global NPU program may have been particularly influenced by the median of 21.1 months among patients from Taiwan [12], who were not included in the global NPU analysis [4]. However, the prolonged outcomes reported here for patients with G719X, L861Q, or S768I mutations are in agreement with data from the LUX-Lung 2, 3, and 6 studies; for patients with G719X, L861Q or S768I, ORR ranged from 56 to 100%, median PFS 8–15 months, and median OS was 17 months–not estimated. The authors concluded that afatinib has activity against NSCLC tumors harboring these types of uncommon *EGFR* mutations [3].

Compared with patients with other *EGFR* mutations, patients with *EGFR* ex20ins have been reported to respond relatively infrequently to first-generation TKIs [13–15] and have poorer survival outcomes than those with common *EGFR* mutations [16]. One review of 84 patients with ex20ins (based on eight small series of between 2 and 25 patients) treated with gefitinib or erlotinib found a response rate of 11% [17]. In another study, median TTF for 8 patients with *EGFR* ex20ins taking erlotinib for advanced disease was 2.4 months, compared with 5.9 months for 17 patients taking combination chemotherapy [16]. In the LUX-Lung 2, 3, and 6 trials, responses to afatinib were least common in patients with ex20ins, with two of 23 patients (8.7%) having an objective response [3]. Afatinib did not confer clinical benefit in these patients. Median PFS was 2.7 months (*n* = 23), which was similar to the PFS of 2.9 months in 14 patients with *de-novo* T790M resistance mutations [3]. Based on the evidence to date, NSCLC patients harboring *EGFR* ex20ins have been described as a unique subset, for whom there are no effective approved targeted therapies [15].

In terms of response to treatment, our findings for patients with ex20ins are similar to previous reports. In this study, 1/5 patients with ex20ins (20%) exhibited PR with afatinib, with 7/20 patients (35%) in the analysis of the global NPU program [4]. The median TTF for 23 patients with *EGFR* ex20ins in this study, however, was 18.9 months, longer than for any other subgroup in this analysis, and much longer than the median of 3.6 months for patients with ex20ins in the global NPU program (*n* = 57) [4]. This disparity may be explained, in part, by the presence of clinical and biochemical heterogeneity between different types of *EGFR* ex20ins, which actually represent a broad subtype of *EGFR* mutations. Kosaka et al. [15] identified at least 19 different types of ex20ins [15], which differed markedly in their susceptibility to afatinib in vitro. While most ex20ins are resistant to EGFR inhibitors, one appears to confer sensitivity to erlotinib (A763_Y764insFQEA) [18, 19]. There is also evidence that the nature of the ex20ins can influence sensitivity to afatinib. A patient in the NPU program with an *EGFR* ex20ins (A767_S768insSVA tandem duplication) was maintained at minimal PD for 54 months and received afatinib for 36 months [20]; this raises the possibility that some patients with *EGFR* ex20ins may obtain benefit from afatinib in this setting. Recently, Liu et al. [21] identified ex20ins in 2% of Chinese patients with lung cancer (171/7,520); detailed survival information during afatinib monotherapy was available for 19 patients. Those harboring G778_P780dup (G778) had longer PFS (median 10 vs. 3.3 months, *p* = 0.32) and OS (median 19.7 vs. 7 months, *p* = 0.16) than those with other ex20ins [21]. A limitation of the current analysis is that ex20ins type was not identified for most patients. This may be important for the correct interpretation of therapeutic outcomes in patients with ex20ins and should be considered in future studies.

All 12 patients who were *HER2* mutation-positive were treated at centers in Taiwan, and all specified *HER2* mutations were in exon 20. Most *HER2* mutations are in exon 20, occurring in 2–4% of patients with NSCLC [22–25]. In the current analysis, median TTF in this subgroup was 12.2 months, and 12.4 months in the 7 patients with the p.A775_G776insYVMA *HER2* mutation. For the 28 *HER2* mutation-positive patients in the global NPU, the median TTF was 2.9 months, but eight of the 28 patients (29%) had TTF > 1 year. The most frequent *HER2* mutation was p.A775_G776insYVMA (insertion at nucleotide 2325), which was identified in 10 of the 12 patients with specified *HER2* mutations (83%). The median TTF for these patients was 9.6 months [9].

During the NPU program, the third-generation EGFR TKI osimertinib was approved for patients with *EGFR* T790M-mutation-positive NSCLC, following acquired resistance to EGFR TKIs. In a phase II study, the ORR of osimertinib in pretreated T790M-positive NSCLC patients was 62% [26], higher than reported here (25%). Osimertinib has also been reported to achieve a high response rate in patients with the uncommon L861Q and G719A/C/D/S/X *EGFR* mutations [27]. The effects of osimertinib treatment in patients with other uncommon *EGFR* mutations have not been reported but, given its specificity for EGFR/ErbB1, unlike afatinib, osimertinib would not be expected to confer benefits in patients with *HER2* mutations.

The safety data observed across the Asian centers were consistent with those reported from non-Asian centers in the NPU program [4, 7, 8] and with the known safety profile of afatinib. Several of the more frequently reported events were not considered to be side effects of afatinib but were symptoms or consequences of advanced-stage lung cancer or disease progression (e.g. fatigue/asthenia, pneumonia, pleural effusion, and cough).

The current analysis has some limitations. The data generated by the NPU program were derived from patients treated in accordance with local clinical practices, but data collection relied on voluntary reporting of patient information by the investigators. Consequently, the proportions of patients with data available on tumor histology, *EGFR* mutation type, response and TTF were relatively low. Also, physicians may have been less spontaneous in providing information on response if their patients did not show a significant therapeutic response; this could have led to under-reporting of patients who did worse, and overestimation of ORR and TTF. Centralized radiographic confirmation of tumor response/SD was not available. The data may also have been influenced by failures to report certain end dates, such as end of treatment and date of death. Additionally, AEs may have been under-reported in this real-world setting compared with the rates of reporting generally seen in clinical trials. Finally, information on *EGFR* mutation status was available for 57% of the patients, but the validation of the results by a central laboratory was not available. No information was available on the timing of the mutation analysis, particularly in relation to the timing of first-generation TKI therapy. Nevertheless, this afatinib NPU program provided practical data on the safety and efficacy of afatinib in the setting of local clinical practices in diverse Asian countries, while facilitating compassionate use of afatinib in a large group of NSCLC patients who had exhausted all other treatment options.

## Conclusions

The findings of the current analysis of data from Asian centers and those of the global NPU [4] were generally similar. Afatinib achieved clinical benefits in patients with refractory NSCLC, both with common and uncommon *EGFR* mutations. In patients with tumors harboring uncommon *EGFR* mutations, estimates of ORR and TTF were similar, if not superior, to those in patients with common *EGFR* mutations. The variation in the efficacy of afatinib treatment in NSCLC harboring different uncommon mutations remains an important area for future research.

## Data Availability

To ensure independent interpretation of clinical study results, Boehringer Ingelheim grants all external authors access to all relevant material, including participant-level clinical study data, and relevant material as needed by them to fulfill their role and obligations as authors under the ICMJE criteria. Furthermore, clinical study documents (e.g. study report, study protocol, statistical analysis plan) and participant clinical study data are available to be shared after the publication of the primary manuscript in a peer-reviewed journal and if regulatory activities are complete and other criteria met per the BI Policy on Transparency and Publication of Clinical Study Data: https://trials.boehringer-ingelheim.com/transparency_policy.html. Prior to providing access, documents will be examined, and, if necessary, redacted and the data will be de-identified, to protect the personal data of study participants and personnel, and to respect the boundaries of the informed consent of the study participants. Clinical Study Reports and Related Clinical Documents can be requested via this link: https://trials.boehringer-ingelheim.com/trial_results/clinical_submission_documents.html. All such requests will be governed by a Document Sharing Agreement. Bonafide, qualified scientific and medical researchers may request access to de-identified, analyzable participant clinical study data with corresponding documentation describing the structure and content of the datasets. Upon approval, and governed by a Data Sharing Agreement, data are shared in a secured data-access system for a limited period of 1 year, which may be extended upon request. Researchers should use https://trials.boehringer-ingelheim.com to request access to study data.
